# Pleomorphic archaeal viruses: the family *Pleolipoviridae* is expanding by seven new species

**DOI:** 10.1007/s00705-020-04689-1

**Published:** 2020-06-24

**Authors:** Tatiana A. Demina, Hanna M. Oksanen

**Affiliations:** Molecular and Integrative Biosciences Research Programme, Faculty of Biological and Environmental Sciences, University of Helsinki, Helsinki, Finland

## Abstract

Established in 2016, the family *Pleolipoviridae* comprises globally distributed archaeal viruses that produce pleomorphic particles. Pseudo-spherical enveloped virions of pleolipoviruses are membrane vesicles carrying a nucleic acid cargo. The cargo can be either a single-stranded or double-stranded DNA molecule, making this group the first family introduced in the 10^th^ Report on Virus Taxonomy including both single-stranded and double-stranded DNA viruses. The length of the genomes is approximately 7–17 kilobase pairs, or kilonucleotides in the case of single-stranded molecules. The genomes are circular single-stranded DNA, circular double-stranded DNA, or linear double-stranded DNA molecules. Currently, eight virus species and seven proposed species are classified in three genera: *Alphapleolipovirus* (five species)*, Betapleolipovirus* (nine species), and *Gammapleolipovirus* (one species). Here, we summarize the updated taxonomy of the family *Pleolipoviridae* to reflect recent advances in this field, with the focus on seven newly proposed species in the genus *Betapleolipovirus*: *Betapleolipovirus HHPV3, HHPV4, HRPV9, HRPV10, HRPV11, HRPV12*, and *SNJ2*.

## Introduction

Archaeal viruses have unique morphological and genetic features as well as prominent roles in global ecological processes [14, 26]. Among viruses infecting halophilic euryarchaeotes, pleomorphic ones represent the second largest group after tailed icosahedral viruses [14, 26]. Currently, a decade after the discovery of the first pleomorphic archaeal virus, Halorubrum pleomorphic virus 1 (HRPV-1), in 2009 [21], 18 such virus isolates are known [4, 5, 9, 17, 19]. Here, following the accumulation of new data, descriptions of new pleolipovirus isolates, and the recent submission of taxonomical proposals [10, 20], we provide an updated overview of this group of viruses, describing their life cycle, host range, virion components, and genomic content.

## The family *Pleolipoviridae*

**Fig. 1:**
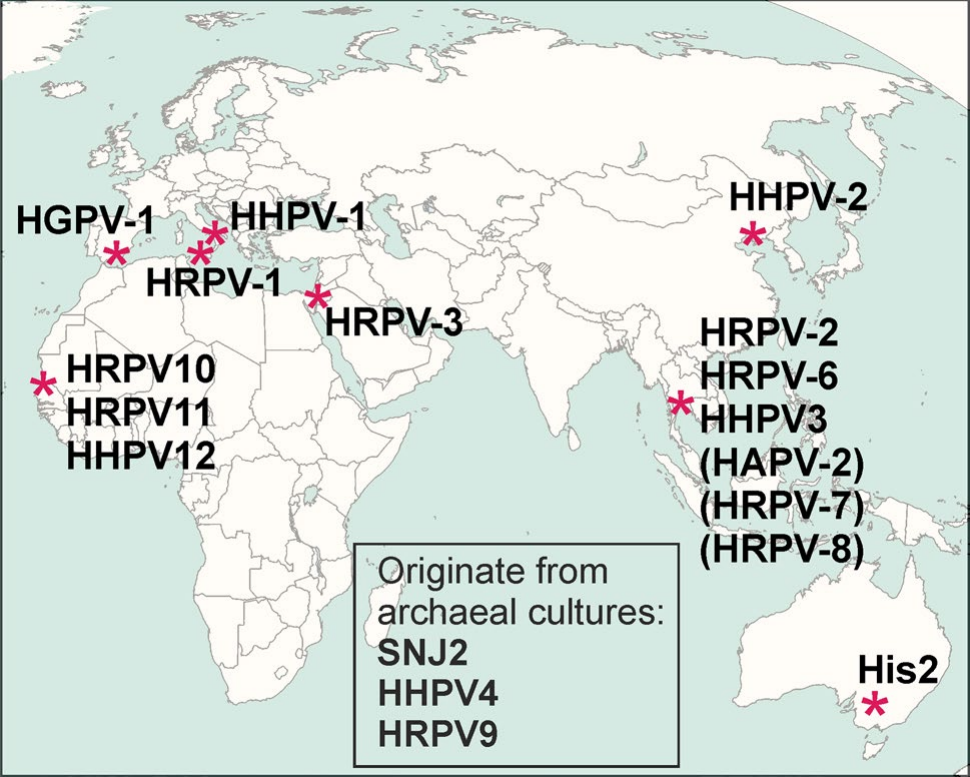
Sites from which pleolipoviruses have been isolated (see also Table 1). Haloarcula hispanica pleomorphic virus 4 (HHPV4), Halorubrum pleomorphic virus 9 (HRPV9), and Saline Natrinema sp. J7-1 virus 2 (SNJ2) originate from archaeal cultures. Haloarcula pleomorphic virus 2 (HAPV-2), Halorubrum pleomorphic virus 7 (HRPV-7), and Halorubrum pleomorphic virus 8 (HRPV-8) shown in brackets are currently unclassified. Original map source: Wikimedia Commons

Pleomorphic archaeal viruses have been isolated from various hypersaline environments all over the world (Table 1, Fig. 1). The currently described pleolipoviruses have been isolated on halophilic archaeal strains belonging to the genera *Halorubrum*, *Haloarcula, Halogeometr****ic****um,* and *Natrinema* of the class Halobacteria. Pleolipoviruses establish non-lytic infection, presumably using budding as an exit mechanism. Virus particles are produced continuously, and the infection may slightly retard host growth (e.g., Halogeometricum pleomorphic virus 1 [HGPV-1]) [23] or impair it significantly (e.g., His2) [30]. As shown for His2, despite the growth retardation, host membranes remain intact during viral egress, as no ATP leakage or lowered oxygen consumption has been observed in infected cells [30]. Saline Natrinema sp. J7-1 virus 2 (SNJ2) is the only temperate pleolipovirus that has been described. The host range of pleolipoviruses is typically very narrow, often limited to the specific isolation host (Table 1). Some pleolipoviruses have been shown to tolerate a wide range of NaCl concentrations, while others are more sensitive to lowered levels of NaCl or other ions [9, 23].

**Table 1:** Members of the family *Pleolipoviridae* and related unclassified virus isolates

Genus	Virus species^b^	Virus isolate and its abbreviation	Isolation host	Other hosts supporting plaque formation	Isolation source	Virion diameter, nm	Genome topology, size, and GenBank accession no.	Reference
*Alphapleolipovirus*	*Alphapleolipovirus HRPV1*	Halorubrum pleomorphic virus 1 (HRPV-1)	*Halorubrum* sp. PV6	-^d^	Saltern, Trapani, Italy	41	Circular ssDNA 7,048 nt FJ685651	[21–23]
	*Alphapleolipovirus HRPV2*	Halorubrum pleomorphic virus 2 (HRPV-2)	*Halorubrum* sp. SS5-4	-	Saltern, Samut Sakhon, Thailand	54	Circular ssDNA 10,656 nt JN882264	[1, 23, 28]
	*Alphapleolipovirus HRPV6*	Halorubrum pleomorphic virus 6 (HRPV-6)	*Halorubrum* sp. SS7-4	-	Saltern, Samut Sakhon, Thailand	49	Circular ssDNA 8,549 nt JN882266	[23, 28]
	*Alphapleolipovirus HHPV1*	Haloarcula hispanica pleomorphic virus 1 (HHPV-1)	*Haloarcula hispanica*	-	Saltern, Margherita di Savoia, Italy	52	Circular dsDNA 8,082 bp GU321093	[1, 27, 28]
	*Alphapleolipovirus HHPV2*	Haloarcula hispanica pleomorphic virus 2 (HHPV-2)	*Haloarcula hispanica*	N/d	Saltern, Hulu Island, Liaoning, China	50	Circular ssDNA 8,176 nt KF056323	[16]
*Betapleolipovirus*	*Betapleolipovirus HRPV3*	Halorubrum pleomorphic virus 3 (HRPV-3)	*Halorubrum* sp. SP3-3	-	Salt water, Sedom Ponds, Israel	67	Circular dsDNA 8,770 bp JN882265	[1, 23, 28]
	*Betapleolipovirus HGPV1*	Halogeometricum pleomorphic virus 1 (HGPV-1)	*Halogeometricum* sp. CG-9	-	Saltern, Cabo de Gata, Spain	56	Circular dsDNA 9,694 bp JN882267	[1, 23, 28]
	*Betapleolipovirus HRPV10* ^*c*^	Halorubrum pleomorphic virus 10 (HRPV10)	*Halorubrum* sp. LR2-17	*Halorubrum* sp. LR2-12	Lake Retba, Senegal	55	Circular dsDNA 9,296 bp MG550111	[19]
	*Betapleolipovirus HRPV11* ^*c*^	Halorubrum pleomorphic virus 11 (HRPV11)	*Halorubrum* sp. LR2-12	*Halorubrum* sp. LR1-15, *Halorubrum* sp. LR1-21, *Halorubrum* sp. LR2-13, *Halorubrum* sp. E200-4	Lake Retba, Senegal	55	Circular dsDNA 9,368 bp MG550113	[19]
	*Betapleolipovirus HRPV12* ^*c*^	Halorubrum pleomorphic virus 12 (HRPV12)	*Halorubrum* sp. LR1-23	*Halorubrum* sp. LR2-12	Lake Retba, Senegal	55	Circular dsDNA 9,944 bp MG550110	[19]
	*Betapleolipovirus HHPV3* ^*c*^	Haloarcula hispanica pleomorphic virus 3 (HHPV3)	*Haloarcula hispanica*	-	Saltern, Samut Sakhon, Thailand	50	Circular dsDNA 11,648 bp KX344510	[3, 9]
	*Betapleolipovirus HHPV4* ^*c*^	Haloarcula hispanica pleomorphic virus 4 (HHPV4)	*Haloarcula hispanica*	N/d	Culture supernatant of *Haloferax* sp. s5a–1 on *Har. hispanica* culture	60	Circular dsDNA 15,010 bp KY264020	[5]
	*Betapleolipovirus HRPV9* ^*c*^	Halorubrum pleomorphic virus 9 (HRPV9)	*Halorubrum* sp. SS5-4	*Halorubrum* sp. SS7-4	Culture supernatant of *Halorubrum* sp. B2–2 on *Halorubrum* sp. SS5-4 culture	57	Circular dsDNA 16,159 bp KY965934	[2, 4]
	*Betapleolipovirus SNJ2* ^*c*^	Saline Natrinema sp. J7-1 virus 2 (SNJ2)	Induced from *Natrinema* sp. J7-1	Can be induced from *Natrinema* sp. J7-2 and *Natrinema* sp. CJ7	*Natrinema* sp. J7-1 culture	70-80	Circular dsDNA 16,992 bp AJVG01000023 (WGS contig04, 19,792-36,797)	[17, 30]
*Gammapleolipovirus*	*Gammapleolipovirus His2*	His2	*Haloarcula hispanica*	-	Pink Lakes, Victoria, Australia	71	Linear dsDNA 16,067 bp AF191797	[7, 25, 28]
N/d^a^	N/d	Haloarcula pleomorphic virus 2 (HAPV-2)	*Haloarcula* sp. SS13-14	-	Saltern, Samut Sakhon, Thailand	N/d	N/d	[3]
N/d	N/d	Halorubrum pleomorphic virus 7 (HRPV-7)	*Halorubrum* sp. SS5-4	*Halorubrum* sp. SS7-4	Saltern, Samut Sakhon, Thailand	N/d	N/d	[3]
N/d	N/d	Halorubrum pleomorphic virus 8 (HRPV-8)	*Halorubrum* sp. SP3-3	*Halorubrum* sp. SS8-2	Saltern, Samut Sakhon, Thailand	N/d	N/d	[3]

^a^N/d, not determined or not reported

^b^Virus species names are given according to the proposal to rename the species in the family *Pleolipoviridae* by using binomial species names [20]

^c^Proposed species

^d^None of the tested strains. For details, see original publications

**Fig. 2:**
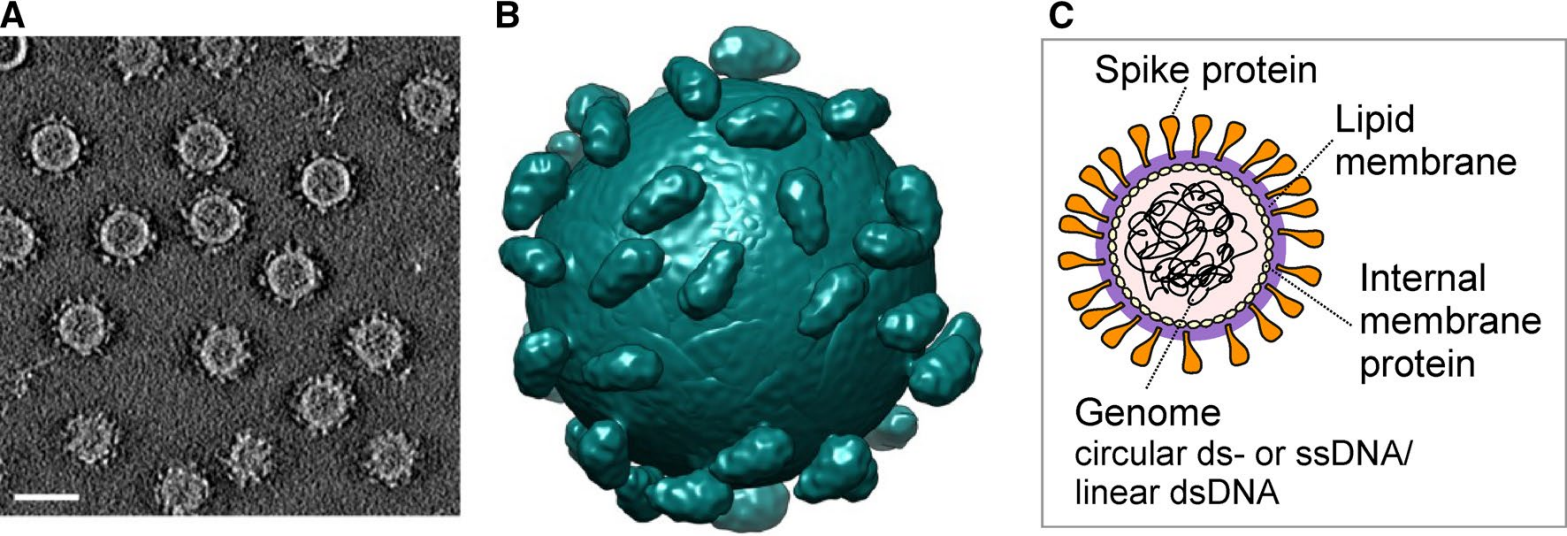
Pleolipoviral virion. (A) Cryo-electron tomography of Halorubrum pleomorphic virus 6 (HRPV-6). A slice through a tomogram of HRPV-6. The bar is 40 nm. (B) Structure of HRPV-6 virion based on the tomography. (C) Schematic overview of pleolipoviral virion. Sections A and B are reproduced from reference 12

Pleolipoviruses lack a rigid protein capsid. Instead, their virions are simple, spherical, and flexible membrane vesicles of 40-80 nm in diameter [4–6, 9, 16, 17, 19, 23, 24] (Fig. 2). They have only 2-4 major structural protein types, either located at the inner side of the membrane or irregularly distributed on the virion surface as spikes (Fig. 2) [4, 5, 9, 17, 19, 23]. Lipids forming the viral membrane vesicle are obtained non-selectively from the host membrane during virion assembly [4–6, 9, 17, 19, 23, 24]. Recent crystallographic structures of Halorubrum pleomorphic virus 2 (HRPV-2) and Halorubrum pleomorphic virus 6 (HRPV-6) spike proteins showed that the monomeric pleolipoviral spike proteins undergo conformational changes and induce the fusion of the viral and host membranes. The spike protein is composed of two roughly equal domains, and its so-called V-shape represents a unique fold. The pre-fusion form of the spike protein is extended to an elongated conformation while being inserted into the membrane to initiate fusion [12].

**Fig. 3:**
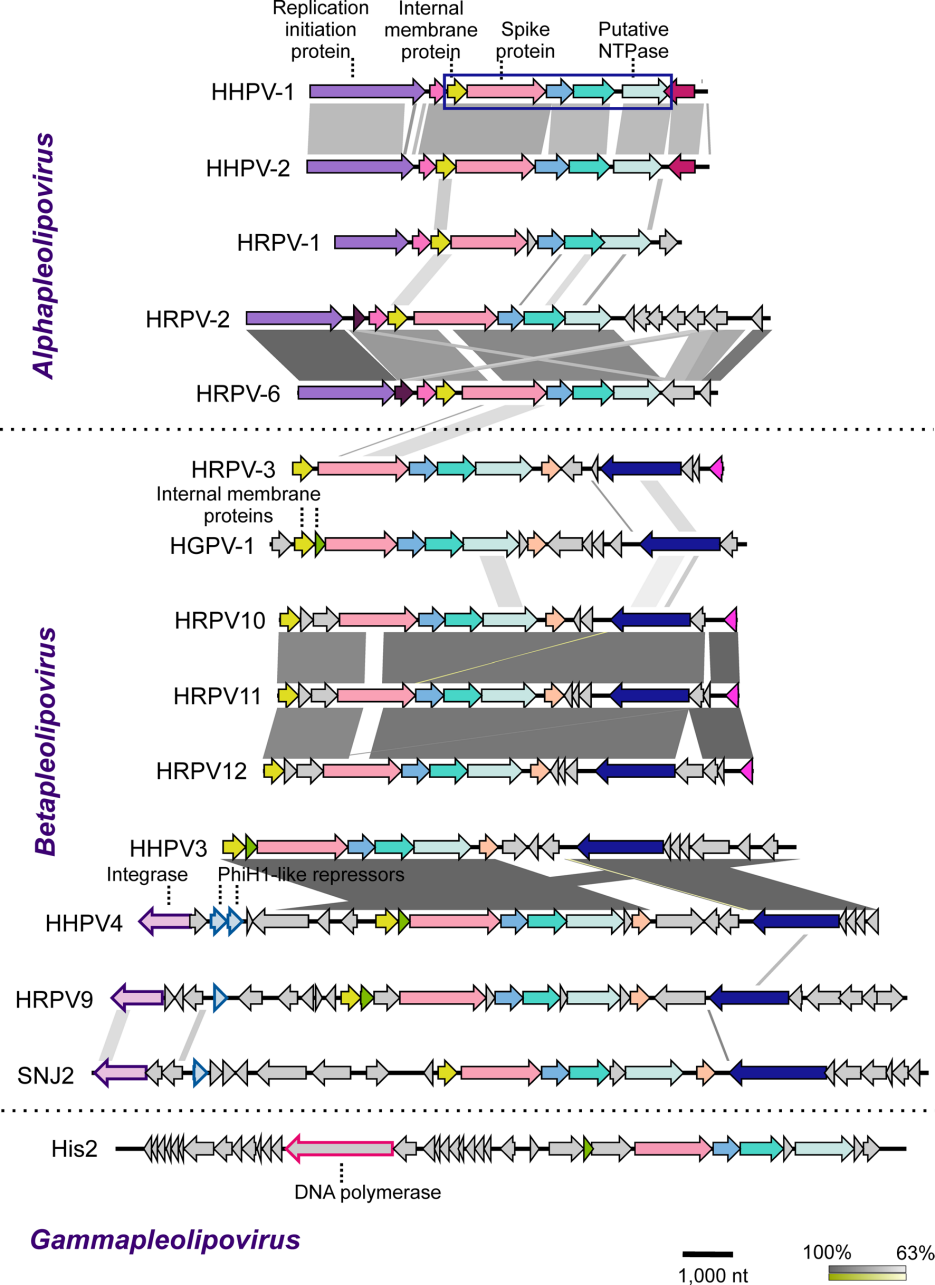
Linear representation of the pleolipovirus genomes and the division of the viruses into the three genera in the family *Pleolipoviridae* shown on the left. Genes and ORFs are shown as arrows, while the shading between them represents the percentage of nucleotide sequence identity (BLASTn, max. e-value 0.001) from 63% to 100% (shades of grey or green for direct and inverted similarities, respectively, see lower panel). Similar genes and ORFs are highlighted with the same colours. In Haloarcula hispanica pleomorphic virus 1 (HHPV-1), the box shows the conserved block of genes encoding the spike protein and integral membrane protein as well as ORFs with no assigned functions that are shared by all pleolipoviruses. The image was generated using Easyfig version 2.2.3

Pleolipoviruses have circular single-stranded (ss) or double-stranded (ds) DNA genomes, except His2, whose genome is a linear dsDNA molecule (Table 1). Genome length ranges from ~7 to ~17 kilobase pairs or kilonucleotides. The dsDNA genomes of viruses belonging to the genus *Betapleolipovirus* may be interrupted by single-stranded regions, whose function is unclear [5, 9, 17, 19, 28]. All pleolipovirus genomes carry a conserved block of collinear genes, encoding structural proteins, a putative NTPase, and other putative proteins (Fig. 2) [4, 19, 28]. The sequence identity between pleolipovirus genomes is typically low, with the following exceptions: The overall nucleotide sequence identity between Haloarcula hispanica pleomorphic virus 1 (HHPV-1) and Haloarcula hispanica pleomorphic virus 2 (HHPV-2) is ~71% [16], as is the case with HRPV-2 and HRPV-6 [28]. Halorubrum pleomorphic virus 10 (HRPV10), Halorubrum pleomorphic virus 11 (HRPV11), and Halorubrum pleomorphic virus 12 (HRPV12), recently isolated from Lake Retba, also have highly similar genomes with 92-95% nucleotide sequence identity [19]. Furthermore, Haloarcula hispanica pleomorphic virus 3 (HHPV3) and Haloarcula hispanica pleomorphic virus 4 (HHPV4) share identical genomic regions (Fig. 3) [5]. In addition, pleolipovirus-related proviral regions commonly found in the haloarchaeal genomes may share up to 100% identity with viral genes [4, 7–9, 11, 17, 19, 21, 27, 28]. Integrase genes are present in the HHPV4, SNJ2, and Halorubrum pleomorphic virus 9 (HRPV9) genomes (see below) [4, 5, 17].

## Current taxonomy of the family *Pleolipoviridae*

The family *Pleolipoviridae* currently has three genera containing fifteen species either approved (8 species) or newly proposed (7 species). The genera, *Alpha-*, *Beta-*, and *Gammapleolipovirus,* are distinguished based on the whole-genome sequence identity and gene content of their members (Table 1, Fig. 3) [6]. In addition to the conserved block of genes shared by all pleolipoviruses, the members of the three genera have specific open reading frames (ORFs). Alphapleolipoviruses encode a putative rolling-circle replication initiation protein, while the only described gammapleolipovirus, His2, encodes a putative protein-primed DNA polymerase, suggesting rolling-circle and protein-primed replication modes, respectively [28]. The linear dsDNA genome of His2 also has inverted terminal repeats and terminal proteins attached at the ends [7, 25]. Betapleolipoviruses have two additional conserved ORFs downstream of the conserved block not found in other pleolipoviruses [4, 19, 28]. One of these (HGPV-1 ORF14 homologue), is suggested to be a replication initiation protein [14, 19]. The genomes of three related pleolipoviruses, Haloarcula pleomorphic virus 2 (HAPV-2), Halorubrum pleomorphic virus 7 (HRPV-7), and Halorubrum pleomorphic virus 8 (HRPV-8) [3], have not been sequenced yet and therefore remain unclassified [6].

## Proposed taxonomic changes in the family *Pleolipoviridae*

The proposals to create seven new species in the genus *Betapleolipovirus* and to change pleolipovirus species names using binomial names consisting of the genus name and the specific epithet have been approved recently by the ICTV Executive Committee [10, 20]. The newly proposed seven species are *Betapleolipovirus HHPV3, HHPV4, HRPV9, HRPV10, HRPV11, HRPV12*, and *SNJ2* (Table 1). Nucleotide sequence identity of ≤95% has been proposed to be the criterion for demarcation of species in the genera of the family *Pleolipoviridae* [10]. The viruses representing the new species and species already in the family *Pleolipoviridae* have collinear dsDNA genomes with ≤95% nucleotide sequence identity shared between two genomes. The genomes of the seven new isolates carry the conserved block of genes and ORFs typical for the members of the family *Pleolipoviridae*, as well as two ORFs specific to the members of genus *Betapleolipovirus* [4, 5, 9, 17, 19].

## SNJ2 is the first described temperate pleolipovirus

SNJ2 is the only pleolipovirus described to date for which a temperate life cycle has been demonstrated [17], although there are also other pleolipoviruses with integrase and phiH1-like repressor genes. SNJ2 can be induced from its host strain *Natrinema* sp. J7-1 [17] and is the only pleomorphic virus with *Natrinema* archaea as a host. In the provirus state, the SNJ2 genome is integrated into the host tRNA^Met^ gene, from which it is excised upon the induction of a lytic cycle. The host range of SNJ2 is very narrow, as it is known to infect only *Natrinema* sp. J7 derivatives, which differ by their plasmids (Table 1). The highest production of SNJ2 is observed from *Natrinema* sp. J7-1, which contains the plasmid pHH205, which is a proviral form of the icosahedral internal membrane-containing dsDNA virus SNJ1 (family *Sphaerolipoviridae*), suggesting that SNJ1 promotes the production of SNJ2 [17]. The pleomorphic SNJ2 virions are about 70×80 nm in size and contain two major structural protein species. The SNJ2 lipid composition is similar to that of its host, except that SNJ2 seems to lack phosphatidylglycerophosphate methyl ester, which is present in *Natrinema* sp. J7-1, albeit in low abundance [17].

The SNJ2 genome is a circular dsDNA molecule with single-stranded interruptions associated with a conserved ‘GCCCA’ DNA motif, although the motif is not always followed by the interruption [17]. The same motif has been shown to precede single-stranded interruptions in the genome of the betapleolipovirus Halorubrum pleomorphic virus 3 (HRPV-3) [28]. The SNJ2 genome is 16,992 bp long, has a GC content of 59.1 %, and contains 25 ORFs. A conserved cluster of pleolipoviral genes is present in SNJ2, including ORFs specific to the genus *Betapleolipovirus* (Fig. 3). SNJ2 was the first pleolipovirus known to carry an integrase and a phiH1-like repressor gene in its genome [17]. The SNJ2 repressor has been suggested to function similarly to the repressor originally described in the halophilic archaeal tailed icosahedral virus phiH1, where it is involved in lytic-lysogenic life cycle switches [17, 29]. The SNJ2 integrase excises the virus genome from the host chromosome, but for integration, two small accessory proteins are also needed [17, 31]. The SNJ2-type integrases are widely found in archaeal genomes, being associated with proviruses and other mobile elements [17, 31]. These integrases are suggested to form a distinct family within the tyrosine recombinase superfamily, having five invariant active site residues of the R_I_…K…H_II_XXR_II_…Y pentad (where X is any residue), but with the typical Glu/Asp_I_ and His/Trp_III_ sites substituted by Gly/Ala and Ala/Val residues, respectively [31].

## HHPV3, HHPV4, and HRPV9 survive in a saturated salt

HHPV3 was isolated from the solar saltern of Samut Sakhon, Thailand, on *Haloarcula hispanica* [3]. HHPV4 and HRPV9 were isolated from plaques obtained on *Har. hispanica* and *Halorubrum* sp. SS5-4 lawns, respectively, after the addition of the other archaeal culture supernatant [2, 5] (Table 1). Virus particles efficiently adsorb to their host cells with adsorption rate constants of 2.4 × 10^−12^, 1.7 × 10^−12^, and 8.5 × 10^−11^ ml/min (measured during the first 30 min of infection) for HHPV3, HHPV4, and HRPV9, respectively. After ~2 h of infection, 65-90% of the particles are bound to cell surfaces. Like other pleolipoviruses, infection with these viruses results in the retardation of the host’s growth [4, 5, 9].

HHPV3 and HHPV4 infectivity drops in the absence of NaCl or CaCl_2_ [5, 9], while for HRPV9, only the absence of NaCl is critical [4]. HHPV3 and HHPV4 show a pH-dependent drop of infectivity at 1-1.5 M NaCl [5, 9], and HRPV9 infectivity is stable over a wide range of NaCl concentrations (0.5-5 M) [4]. HHPV3, HHPV4, and HRPV9, as well as betapleolipoviruses HRPV-3 and HGPV-1, stay infectious even in NaCl-saturated solutions [4, 5, 9], which may be highly beneficial for their survival in hypersaline environments.

The HHPV3, HHPV4, and HRPV9 virus particles are spherical or slightly pleomorphic with a diameter of 50-60 nm [4, 5, 9]. Like HGPV-1, the viruses HHPV3, HHPV4, and HRPV9 have one spike protein and two membrane protein types [4, 5, 9, 23, 28]. Lipid profiles of these viruses resemble those of their hosts, implying non-selective lipid acquisition [4, 5, 9]. The major lipids in HHPV3 and HHPV4 are phosphatidylglycerol, phosphatidylglycerophosphate methyl ester, phosphatidylglycerosulfate, and triglycosyl glycerodiether [5, 9]. The HHPV3, HHPV4, and HRPV9 genomes are circular dsDNA molecules of 11,648, 15,010, and 16,159 bp in length, respectively, with a GC content of 55-60% [4, 5, 9]. Single-stranded interruptions have been suggested in genomes of HHPV3 and HHPV4 [5, 9]. The two viruses have identical genome regions, covering ~65% and ~84% of the whole genome sequences in HHPV4 and HHPV3, respectively. These identical regions include the conserved block of betapleolipoviral genes: all characteristic betapleolipoviral ORFs are 100% identical in HHPV3 and HHPV4, except that HHPV4 ORF16 and HHPV3 ORF7 share ~73% nucleotide sequence identity. The case of HHPV3 and HHPV4 highlights flexibility in pleolipoviral virion assembly, as the larger genome of HHPV4 is packed into vesicles made up of the same major structural components as in HHPV3, which has a smaller genome [5, 9]. Notably, the HHPV4 genome contains a block of an integrase and two phiH1-like repressor genes, which is absent in HHPV3 [5]. The integrase and phiH1-like repressor genes are also found in the HRPV9 genome [4]. The HHPV4 integrase belongs to the SNJ2-type family, while HRPV9 integrase differs, with a Met substitution at the His/Trp_III_ site, where SNJ2-type integrases typically have Ala/Val (see above) [4, 31]. It is unclear whether HHPV4 and HRPV9 integrases are functional or defective [4]. Phylogenomic trees inferred using the Genome-BLAST Distance Phylogeny (GBDP) method suggest that HHPV3, HHPV4, and HRPV9 cluster with SNJ2 and other betapleolipoviruses [4].

## Closely related betapleolipoviruses HRPV10, HRPV11, and HRPV12 from Africa

The most recently discovered viruses, HRPV10, HRPV11, and HRPV12, were isolated from the saline Lake Retba, close to Dakar, Senegal, on *Halorubrum* strains originating from the same location [19]. The viruses produce hazy plaques, typical for pleolipoviruses, suggesting a non-lytic virus life cycle. Out of 19 autochthonous *Halorubrum* strains from Lake Retba that were tested, HRPV11 was found to infect four, while HRPV10 and HRPV12 were capable of infecting only two strains. Out of 51 *Halorubrum* strains from other geographical locations, HRPV11 could infect one strain from Eilat, while the other two viruses had no additional hosts [19]. Thus, these three viruses have narrow and distinct host ranges with a preference for autochthonous strains (Table 1).

HRPV10, HRPV11, and HRPV12 form tailless round virus particles of ~55 nm in diameter, having distinct protein profiles with two major structural proteins. The lipid content seems to be identical in the three viruses, and a non-selective mode of lipid acquisition is suggested. The virus genomes are circular dsDNA molecules with single-stranded regions. The total genome length of HRPV10, HRPV11, and HRPV12, is 9,296, 9,368, and 9,944 bp, respectively, and their GC content is 55.5%. The genomes contain 13-16 ORFs, which are arranged in two putative operons. The three viruses display close genetic similarity, sharing 92-95% overall nucleotide sequence identity, but they are genetically distant from all other pleolipoviruses. In addition to the conserved core genes found in all pleolipoviruses, HRPV10, HRPV11, and HRPV12 have ORFs that are specific to all known betapleolipoviruses (homologues of HRPV10 ORF8 and ORF11) as well as the ORFs conserved in some betapleolipoviruses (HRPV10 ORF10 and ORF13). Phylogenomic trees constructed using the GBDP method show the clustering of these three viruses with HRPV-3, HGPV-1, HHPV3, and SNJ2, i.e., the members of the genus *Betapleolipovirus* [19].

## Conclusions

Concerning virion morphology, pleolipoviruses resemble bacteriophages belonging to the family *Plasmaviridae* [15], although they share no common genes. Deep evolutionary relationships have been proposed between pleolipoviruses and mobile genetic elements, including plasmids enclosed in membrane vesicles [13, 14]. However, the evolutionary pathways are still unclear for this virus group. Having been neglected until 2009, pleomorphic archaeal viruses now seem to be extremely abundant in hypersaline environments all over the planet. The identification of numerous related putative proviruses in the genomes of halophilic archaea that inhabit various locations, including the deep sea [18], also highlights the abundance of pleolipoviruses and related elements. The family *Pleolipoviridae* is expanding fast, and we anticipate that future studies will reveal many more of its members, and/or closely related viruses.

## References

[1] Atanasova NS, Roine E, Oren A, Bamford DH, Oksanen HM (2012). Global network of specific virus-host interactions in hypersaline environments. Environ Microbiol.

[2] Atanasova NS, Pietilä MK, Oksanen HM (2013). Diverse antimicrobial interactions of halophilic archaea and bacteria extend over geographical distances and cross the domain barrier. Microbiol Open.

[3] Atanasova NS, Demina TA, Buivydas A, Bamford DH, Oksanen HM (2015). Archaeal viruses multiply: temporal screening in a solar saltern. Viruses.

[4] Atanasova NS, Demina TA, Krishnam Rajan Shanthi SNV, Oksanen HM, Bamford DH (2018). Extremely halophilic pleomorphic archaeal virus HRPV9 extends the diversity of pleolipoviruses with integrases. Res Microbiol.

[5] Atanasova NS, Heiniö CH, Demina TA, Bamford DH, Oksanen HM (2018). The unexplored diversity of pleolipoviruses: the surprising case of two viruses with identical major structural modules. Genes.

[6] Bamford DH, Pietilä MK, Roine E, Atanasova NS, Dienstbier A, Oksanen HM, ICTV Report Consortium (2017). ICTV virus taxonomy profile: *Pleolipoviridae*. J Gen Virol.

[7] Bath C, Cukalac T, Porter K, Dyall-Smith ML (2006). His1 and His2 are distantly related, spindle-shaped haloviruses belonging to the novel virus group, *Salterprovirus*. Virology.

[8] Chen S, Wang C, Xu JP, Yang ZL (2014). Molecular characterization of pHRDV1, a new virus-like mobile genetic element closely related to pleomorphic viruses in haloarchaea. Extremophiles.

[9] Demina TA, Atanasova NS, Pietilä MK, Oksanen HM, Bamford DH (2016). Vesicle-like virion of *Haloarcula hispanica* pleomorphic virus 3 preserves high infectivity in saturated salt. Virology.

[10] Demina TA, Krupovic M, Oksanen HM (2020) Create seven new species in the genus *Betapleolipovirus*, family *Pleolipoviridae*. International Committee on Taxonomy of Viruses, Taxonomic proposal 2019. https://talk.ictvonline.org/files/proposals/taxonomy_proposals_prokaryote1/m/bact04/9102. Accessed 31 Oct 2019

[11] Dyall-Smith ML, Pfeiffer F, Klee K, Palm P, Gross K, Schuster SC, Rampp M, Oesterhelt D (2011). *Haloquadratum walsbyi*: limited diversity in a global pond. PLoS One.

[12] El Omari K, Li S, Kotecha A, Walter TS, Bignon EA, Harlos K, Somerharju P, De Haas F, Clare DK, Molin M, Hurtado F, Li M, Grimes JM, Bamford DH, Tischler ND, Huiskonen JT, Stuart DI, Roine E (2019). The structure of a prokaryotic viral envelope protein expands the landscape of membrane fusion proteins. Nat Commun.

[13] Erdmann S, Tschitschko B, Zhong L, Raftery MJ, Cavicchioli R (2017). A plasmid from an Antarctic haloarchaeon uses specialized membrane vesicles to disseminate and infect plasmid-free cells. Nat Microbiol.

[14] Krupovic M, Cvirkaite-Krupovic V, Iranzo J, Prangishvili D, Koonin EV (2018). Viruses of archaea: structural, functional, environmental and evolutionary genomics. Virus Res.

[15] Krupovic M, ICTV Report Consortium (2018). ICTV virus taxonomy profile: *Plasmaviridae*. J Gen Virol.

[16] Li M, Wang R, Zhao D, Xiang H (2014). Adaptation of the *Haloarcula hispanica* CRISPR-Cas system to a purified virus strictly requires a priming process. Nucleic Acids Res.

[17] Liu Y, Wang J, Liu Y, Wang Y, Zhang Z, Oksanen HM, Bamford DH, Chen X (2015). Identification and characterization of SNJ2, the first temperate pleolipovirus integrating into the genome of the SNJ1-lysogenic archaeal strain. Mol Microbiol.

[18] Messina E, Sorokin DY, Kublanov IV, Toshchakov S, Lopatina A, Arcadi E, Smedile F, La Spada G, La Cono V, Yakimov MM (2016). Complete genome sequence of ‘*Halanaeroarchaeum sulfurireducens*’ M27-SA2, a sulfur-reducing and acetate-oxidizing haloarchaeon from the deep-sea hypersaline anoxic lake Medee. Stand Genom Sci.

[19] Mizuno CM, Prajapati B, Lucas-Staat S, Sime-Ngando T, Forterre P, Bamford DH, Prangishvili D, Krupovic M, Oksanen HM (2019). Novel haloarchaeal viruses from Lake Retba infecting *Haloferax* and *Halorubrum* species. Environ Microbiol.

[20] Oksanen HM, Krupovic, M. (2020) Rename eight species in the family *Pleolipoviridae*. International Committee on Taxonomy of Viruses, Taxonomical proposal 2019. https://talk.ictvonline.org/files/proposals/taxonomy_proposals_prokaryote1/m/bact04/9100. Accessed 31 Oct 2019

[21] Pietilä MK, Roine E, Paulin L, Kalkkinen N, Bamford DH (2009). An ssDNA virus infecting archaea: a new lineage of viruses with a membrane envelope. Mol Microbiol.

[22] Pietilä MK, Laurinavicius S, Sund J, Roine E, Bamford DH (2010). The single-stranded DNA genome of novel archaeal virus Halorubrum pleomorphic virus 1 is enclosed in the envelope decorated with glycoprotein spikes. J Virol.

[23] Pietilä MK, Atanasova NS, Manole V, Liljeroos L, Butcher SJ, Oksanen HM, Bamford DH (2012). Virion architecture unifies globally distributed pleolipoviruses infecting halophilic archaea. J Virol.

[24] Pietilä MK, Roine E, Sencilo A, Bamford DH, Oksanen HM (2016). *Pleolipoviridae*, a newly proposed family comprising archaeal pleomorphic viruses with single-stranded or double-stranded DNA genomes. Arch Virol.

[25] Porter K, Dyall-Smith ML (2008). Transfection of haloarchaea by the DNAs of spindle and round haloviruses and the use of transposon mutagenesis to identify non-essential regions. Mol Microbiol.

[26] Prangishvili D, Bamford DH, Forterre P, Iranzo J, Koonin EV, Krupovic M (2017). The enigmatic archaeal virosphere. Nat Rev Microbiol.

[27] Roine E, Kukkaro P, Paulin L, Laurinavicius S, Domanska A, Somerharju P, Bamford DH (2010). New, closely related haloarchaeal viral elements with different nucleic acid types. J Virol.

[28] Sencilo A, Paulin L, Kellner S, Helm M, Roine E (2012). Related haloarchaeal pleomorphic viruses contain different genome types. Nucleic Acids Res.

[29] Stolt P, Zillig W (1992). In vivo studies on the effects of immunity genes on early lytic transcription in the *Halobacterium salinarium* phage phi H. Mol Gen Genet.

[30] Svirskaite J, Oksanen HM, Daugelavicius R, Bamford DH (2016). Monitoring physiological changes in haloarchaeal cell during virus release. Viruses.

[31] Wang J, Liu Y, Liu Y, Du K, Xu S, Wang Y, Krupovic M, Chen X (2018). A novel family of tyrosine integrases encoded by the temperate pleolipovirus SNJ2. Nucleic Acids Res.

